# Unveiling the Clinical Spectrum of Post-COVID-19 Conditions: Assessment and Recommended Strategies

**DOI:** 10.7759/cureus.52827

**Published:** 2024-01-23

**Authors:** Abdullah M Assiri, Tareef Alamaa, Faisal Elenezi, Aeshah Alsagheir, Lamya Alzubaidi, Imad TIeyjeh, Abdulazia S Alhomod, Eisha M Gaffas, Samar A Amer

**Affiliations:** 1 Preventive Services, Saudi Ministry of Health, Riyadh, SAU; 2 Therapeutic Services, Saudi Ministry of Health, Riyadh, SAU; 3 Assistant Agency for Hospital Affairs, Saudi Ministry of Health, Riyadh, SAU; 4 Health Quality Index Measuring, Saudi Ministry of Health, Riyadh, SAU; 5 Infectious Diseases, Mayo Clinic, Rochester, USA; 6 Infectious Diseases, King Fahad Medical City, Riyadh, SAU; 7 Seha Virtual Hospital, Saudi Ministry of Health, Riyadh, SAU; 8 Mental Health and Social Services, Saudi Ministry of Health, Riyadh, SAU; 9 Public Health and Community Medicine, Zagazig University, Zagazig, EGY; 10 General Administration of Health Programs and Non-communicable Diseases, Saudi Ministry of Health, Riyadh, SAU

**Keywords:** post-covid-19 conditions (pccs), education management solutions, clinical assessment tools, clinical symptoms, review article

## Abstract

SARS-CoV-2 caused the pandemic of the rapidly evolving COVID-19. As of December 6, 2023, there were 765,152,854 COVID-19-recovering cases. Long-term consequences known as "long COVID" and "post-COVID-19 conditions" (PCCs) or "post-acute COVID-19 syndrome" are being reported more frequently in a subset of recovering patients. Systemic, neuropsychiatric, cardio-respiratory, and gastrointestinal symptoms are the most prevalent.

The management of PCCs poses unique challenges due to the lack of official guidelines and the complex nature of the illness. This abstract highlights key principles derived from recent reviews and expert recommendations to provide healthcare professionals with a comprehensive approach to manage post-COVID-19 patients.

Preventive medicine plays a crucial role in managing PCCs. While no specific medications are available for treatment, preventive measures such as COVID-19 vaccination, adherence to precautionary measures, regular consultations with medical professionals, monitoring symptoms and progress, and seeking information on symptom management are essential to assist patients in their recovery and improve their quality of life.

Medical management requires transparent goal-setting and collaborative decision-making based on the patient's symptoms, comorbidities, and treatment objectives. Treatment plans for post-COVID-19 patients should focus on patient education, using registries and calendars to track symptoms and triggers, providing support and reassurance, and offering holistic support through peer networks and supportive psychotherapy techniques. Symptomatic and rehabilitative care, including well-established symptom management techniques, physical rehabilitation programs, and addressing mental health and well-being, are vital components of post-COVID-19 management.

Lifestyle factors such as stress reduction, nutrition, and sleep should be incorporated into managing underlying medical conditions in post-COVID-19 patients. Regular follow-up visits and referrals to specialists are recommended to monitor the patient's progress and address specific organ system involvement or additional care needs.

In summary, for the effective management of PCCs, a holistic approach should include preventive measures, patient education, supportive psychotherapy, symptomatic and rehabilitative care, medical management, counseling on lifestyle elements, and appropriate follow-up plans. However, it is crucial to stay updated with evolving guidelines and recommendations from healthcare authorities to provide the most effective and evidence-based care to post-COVID-19 patients.

## Introduction and background

SARS-CoV-2 caused the pandemic of the rapidly evolving COVID-19. As of December 6, 2023, there were 765,152,854 COVID-19 recovering cases [1]. Long-term consequences are known as various alternative terms used to describe the post-COVID-19 conditions (PCCs), including "long-COVID," "post-COVID-19 syndrome," "chronic COVID syndrome," "late sequelae of COVID-19," "long-haul COVID," "long-term COVID-19," "post-acute COVID-19 infection," and "COVID long-haulers" [2-3]. PCCs encompass a diverse range of signs and symptoms that persist or worsen for more than 12 weeks following an acute SARS-CoV-2 infection. These symptoms can manifest individually or in various combinations, often overlapping, and may be transient, constant, or fluctuating over time, affecting any system of the body. While PCCs represent an ongoing illness, there is currently no globally accepted definition for this condition [3-4].

Accurately estimating the number of COVID-19 cases with PCCs is challenging due to several factors: variations in diagnostic precision, population size, healthcare capacity, and methodology contribute to discrepancies in the epidemiological reports of PCCs [5]. The prevalence of PCC symptoms is approximately 2% among adolescents and 50% among adults worldwide [6]. The duration of PCCs depends on the length of follow-up, the specific symptoms under investigation, and the accuracy of self-reporting. For instance, studies have shown that 33% of patients had not returned to their normal state of health three to six weeks after diagnosis, while figures ranged from 32.6% to 87% at 60 days, 96% at 90 days, 76% at six months, and 30% at nine months. It's worth noting that 85% of the respondents in these studies were outpatients with milder conditions [7-8]. The United Kingdom Office for National Statistics has reported that one in 10 COVID-19 cases may experience symptoms for three months or longer, with 22% still experiencing symptoms after five weeks and 9.9% at three months. In the United States, patients showed over 32% had experienced new-onset symptoms or were persisting; more than 15% required re-hospitalization with more than 6% of mortality [9-10].

The onset patterns of PCCs can be categorized as follows: (1) persistent conditions that arise at the onset of acute infection, (2) new-onset and late sequelae that emerge after the asymptomatic phase or the resolution of acute symptoms, and (3) evolving symptoms and conditions characterized by the addition of new symptoms over time (e.g., memory loss) alongside the persistence of some initial symptoms (e.g., dyspnea) during the COVID-19 disease or the relief period following acute symptoms [11-14].

Despite the significant efforts to explain the different symptoms of PCCs, current knowledge is still limited. Moreover, according to our knowledge, no single review or study covers all aspects related to the PCCs. Therefore, we conducted this comprehensive review to explore the estimated prevalence, symptoms, underlying pathophysiological mechanisms, standard clinical assessments, laboratory tests, imaging, coding, and suggested management strategies for conditions linked to the most prevalent PCCs, like fatigue, musculoskeletal and pulmonary disorders, cardiovascular problems, neurological impairments, gastrointestinal and hepatic disorders, urology and renal disorders, mental, metabolic, and endocrine disorders, ear, nose, and throat disorders, dermatological disorders, and hematological disorders.

## Review

Methods

This review aims to provide healthcare professionals with updated knowledge, skills, and tools to effectively diagnose and manage PCCs based on current evidence for best practice. The reviews target the following healthcare professionals: physicians, psychologists, physiotherapists, public health workers, laboratory technicians, nurses, and other professionals working in primary and secondary healthcare settings. This review addresses six critical elements, namely patient population, intervention, comparison, outcome, and setting (PICOS), to define and cover various aspects. In terms of the patient population (P), the focus is on the target population and the characteristics of the disease condition. Specifically, the review pertains to patients meeting the clinical case definition of COVID-19 and PCCs. Regarding interventions (I), the review delves into screening, diagnosing, and assessing the population for long-term COVID-19 cases, management interventions, and referrals. The comparison aspect (C) involves evaluating appropriate interventions and management plans and selecting the optimal measurement tool. Anticipated outcomes (O) include the reduction of burdens associated with PCCs (e.g., financial, time, and psychological burden), decreased clinical practice variation, reduced expenditure within the health system, and an overall enhancement in the health status outcome of PCCs. Lastly, the review considers the healthcare setting and the contextual factors (S) in which the guidelines are intended to be implemented.

The development process involved a comprehensive review of all pertinent, scientifically validated literature or articles published in English, addressing the presentation, diagnosis, and treatment of post-COVID-19. Articles focused on subclinical diseases with a veterinary, pediatric, or solely geriatric focus, as well as those about acute COVID-19, were excluded. Following a meticulous evaluation, a decision was made to close the identified gaps and prevent unnecessary duplication of effort. The search terms used are the following: “(long OR post OR 'post-acute') AND (were COVID 19'/exp OR 'COVID 19' OR 'coronavirus infections'/exp OR 'coronavirus infections' OR(('coronavirus'/exp OR coronavirus) AND ('infections'/exp OR infections)) OR 'COVID'/exp 'OR' COVID OR 'sars cov 2'/exp OR'sars cov 2') AND ('fatigue'/'OR' “cardiomyopathies 'OR' cardiomyopathy 'OR' myocarditis”/OR 'musculoskeletal/OR 'respiratory impairments' OR 'dyspnea 'OR' breathlessness 'OR' neurological impairments 'OR' gastrointestinal and hepatic impairment 'OR' psychological impairments and post-traumatic stress disorder 'OR' metabolic impairments 'OR' olfactory impairment 'OR' taste impairment 'OR' cognitive impairments 'OR' menstrual disturbances 'OR' dermatological disorders 'OR' taste impairment). This review included 110 articles after screening more than 750 articles from the three main databases, PubMed, Web of Science, and EMBASE, in September 2023.

COVID-19 Variants of Concern

The SARS-CoV-2 virus belongs to the Coronaviridae subfamily (CoV) of the Coronavirinae family of RNA viruses. Several subfamily COVID-19 variants of concern have emerged, including alpha, beta, gamma, and delta coronaviruses, as summarized in Table 1, exhibiting increased transmissibility and the potential to cause more severe acute illness. As of June 30, 2021, it is crucial to thoroughly investigate whether specific virus strains can lead to long-term consequences. Patients infected with such variants may require additional care and prompt, aggressive treatment approaches to address their long-term symptoms, as certain variants might have more detrimental long-term effects than others [14-16].

**Table 1:** Summary of the COVID-19 variants of concern There are serious worries that the current vaccination methods, which use the wild-type spike (S) protein, may not protect well enough against the quickly spreading variants of concern of SARS-CoV-2 and the gradual loss of antibody immunity. This is because the VOCs have different antigens. The variant includes all of the defined lineages' sub-lineages as well. SARS-CoV-2 and MERS-CoV share 50% of their genomic sequence identity and 79% of their genomic sequence identity. N.B.: Nowadays, no SARS-CoV-2 variants are considered variants of high consequence. x: Encompassing its offspring (BN, CH, and so forth). Under the BA.2.75 lineages, Omicron-Omicron recombinants XBF and XBK that share the same spike as BA.2.75 are observed. y: G257S, D339H, G446S, N460K, Q493 (reversion), W152R, F157L, I210V, and G257S. (a) Keeping an eye on a group of SARS-CoV-2 lineages that share a common set of mutations and comparable spike protein profiles (S: Q183E, S: F486P, and S: F490S). See the table here for the complete list of lineages. (b) Keeping an eye on a group of SARS-CoV-2 lineages (S: F456L, S: Q183E, S: F486P, and S: F490S) that share a common set of mutations and comparable spike protein profiles. See the table here for the complete list of lineages.

Names of the COVID-19 variants of concern	Effect/vaccine efficacy
Wild-type SARS-CoV-2	Which started the epidemic, cough, fever, dyspnea, loss of taste and smell, and diarrhea are symptoms of a lower respiratory tract infection of the wild-type
1^st^ strain is the “Kent variant” from the B.1.1.7 lineage, now termed the Alpha variant	Approximately 40-50% increased transmissibility than the original and likely increased acute disease severity of Oxford AstraZeneca is 74.5%. BioNTech vaccines are 93.7%. Novavax is 85.6%. Moderns are 100%. Sinovac vaccines are 71-91%
Beta, first discovered in May 2020	With a 50% increase in transmission, both Pfizer and Moderna vaccines are still 95% effective. Novavax (60%) and Johnson & Johnson (57%). Oxford-AstraZeneca single-dose vaccines show low efficacy against beta at 82% effectiveness in preventing severe disease and death. Sputnik V’s was 70% lower against beta than against wild-type
Gamma in Manaus, Brazil, in November 2020	The Pfizer vaccine is effective for 60% of fully vaccinated people, while Sputnik V is “highly effective” against gamma variants 1.7-2.4 times more transmissible than wild-type SARS-CoV-2
Delta	The most transmissible form is 60% more so than the alpha variant. 67% with the Oxford-AstraZeneca vaccine and 88% with the Pfizer-BioNTech vaccine, while Sputnik V is 90% effective. First identified in October 2020, is dominant in Europe and the US (9 mutations on the spike protein)
Eta, Zeta, Theta, Lambda, and Kappa variants	Under investigation
Delta-omicron COVID-19 variant	1^st^ discovered on January 9, 2022, 10 of the mutations from Omicron have been found in the new variant
New Omicron (or B.1.1.529) variant (32–50 mutations on his protein)	It is not yet clear whether infection with Omicron causes more severe disease compared to infections with other variants, including Delta. They tended to infect the upper respiratory tract, had higher infectivity, and had reduced pathogenicity in the lungs. symptoms such as headache, sore throat symptoms of an upper respiratory tract infection. Comparing Omicron to Delta and wild-type SARS-CoV-2
Omicron BA.2.75 (x)	Which first was detected in India in May 2022, with a spike mutation in the y, with an unclear impact on transmissibility and severity
Omicron XBB.1.5-like (a)	Which was first detected in the United States, with spike mutations in the N460K, S486P, and F490S, with a similar impact to the baseline on transmissibility, severity, and immunity
Omicron XBB.1.5-like + F456L (b)	With spike mutations in the F456L, N460K, S486P, and F490S, which affected severity and transmissibility that was similar to the baseline, but it had a bigger effect on immunity

Case Definition of PCCs

NICE recommends the use of the term "post-COVID-19 syndrome." Signs and symptoms consistent with COVID-19 that appear during or after infection and persist for more than four weeks are considered long-term COVID-19, provided there is no alternative explanation for the symptoms after 12 weeks of infection. The symptoms, as reported in that review, typically emerge on average between 5 and 20 weeks after the initial infection (Table 2) [17].

**Table 2:** Descriptions of PCCs Image Source: Batiha et al., 2022 [17]

Duration of symptoms	Description/terms
>3 months	Post-COVID-19 syndrome
	Long COVID-19
	Chronic COVID-19
1-3 months	On-going COVID-19
	Post-acute COVID-19
>24 weeks	Persistent post-COVID-19 symptoms
12-24 weeks	Long post-COVID-19 symptoms
5-12 weeks	Acute post-COVID-19 symptoms
>4 weeks (from symptoms onset)	Post-acute COVID-19 syndrome
>4 weeks (from diagnosis time)	Long-COVID-19
	Long-haulers
	Late sequelae of SARS-CoV-2 infection
> 2 months	Long-COVID-19
> 100 days	Long-haul COVID-19

Risk Factors

The risk factors for PCCs are females, advanced age, some pre-existing conditions or co-morbidities, such as a history of neuropsychiatric disorders, type 2 diabetes mellitus, early SARS-CoV-2 RNAemia, reactivation of latent viruses, specifically Epstein-Barr virus, the presence of particular autoantibodies at or before acute COVID-19, and the severity of the initial disease [6,10].

Pathophysiology 

Several mechanisms were suggested to explain the following:

SARS-CoV-2 uses and attaches to an angiotensin-converting enzyme 2 (ACE2) receptor on the cell membrane. ACE2 exhibits a wide distribution and is prominently expressed in various organs, such as the colon, kidney, lung, brain, heart, testis, and certain immune cells.

Severe infection with SARS-CoV-2 leads to an accelerated immune response, immune disturbances, and the release of pro-inflammatory cytokines, resulting in the development of hypercytokinemia and cytokine storms. The virus continues to replicate and proliferate within the body, leading to the activation and infiltration of immune cells, resulting in the release of various cytokines. This immune response can cause hyperinflammation, hypercoagulability, in situ thrombosis, and macrothrombosis.

Research has demonstrated a correlation between the development of mast cell activation syndrome (MCAS) and the progression of post-COVID-19 syndrome (PCS). MACS is a unique type of disorder where mast cells become too active, releasing too many chemical mediators in the wrong places.

Following a critical illness, a complex pathogenesis known as post-intensive care syndrome can manifest. This condition may involve the emergence or progression of anatomical, neurological, and psychological abnormalities. Additional factors such as immobility, metabolic alterations during severe illness, and microvascular ischemia and damage contribute to the risk of developing cardiovascular disease [18-20]

Post-COVID-19 conditions

**Figure 1:**
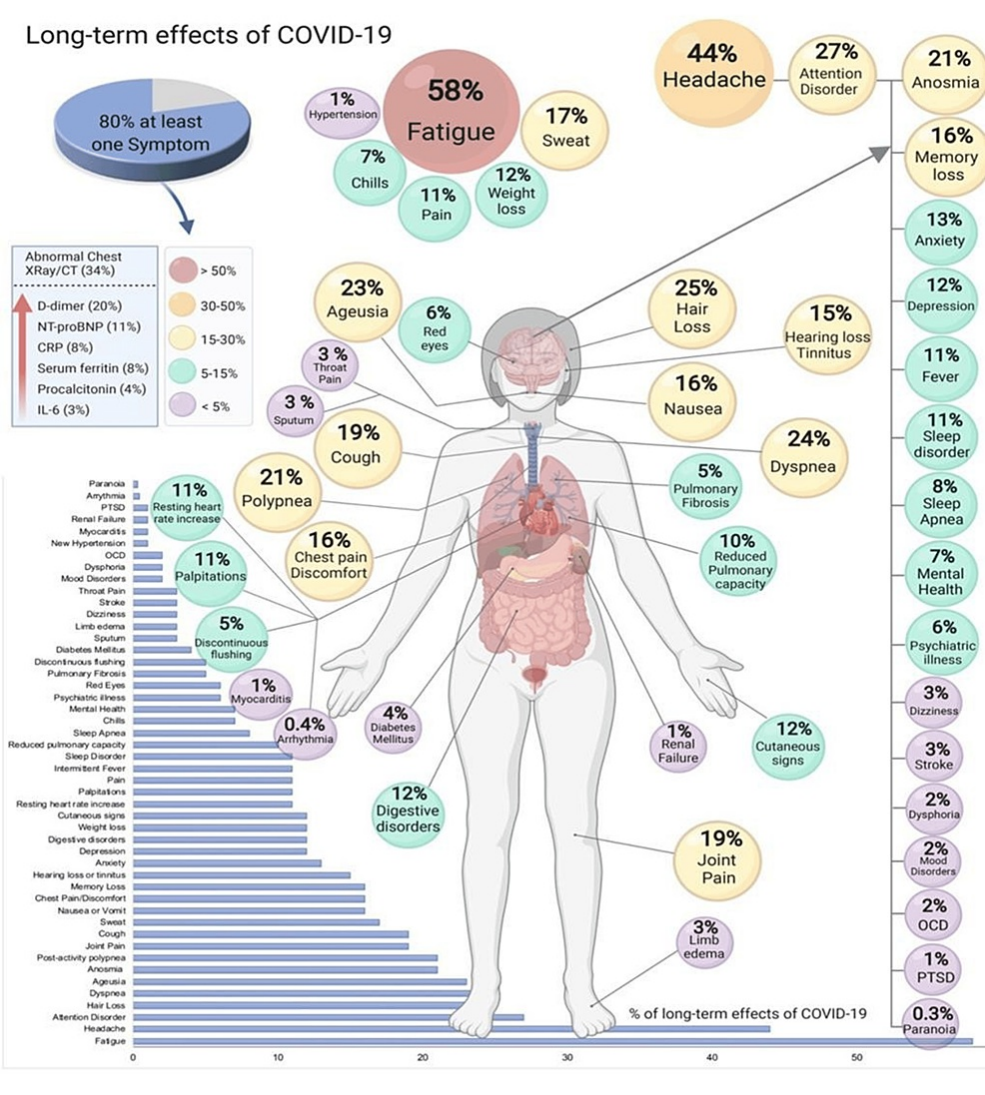
Symptoms and effects of PCCs Image Source: López-León et al., 2021 [22]

PCCs are characterized by functional impairments and encompass various psychological, physical, and social effects that significantly impact the health and overall well-being of the affected individuals. The clinical manifestations of post-acute sequelae of SARS-CoV-2 infection (PASC) across multiple organs have been documented in existing literature, as depicted in Table 2 and Figure 1. These observations highlight the diverse range of clinical consequences associated with PASC [21-30].

Symptoms of PCCs

The most frequently reported symptoms in individuals with PCCs encompass fatigue and shortness of breath. However, there are various additional complaints that individuals may experience. These include, but are not limited to, the following: (1) general symptoms include fatigue, vitamin D deficiency, weight loss, dysautonomias, allergies, MCAS, exacerbation of existing comorbid conditions, reactivation of other viruses, pain syndromes, limitations in activities of daily living (ADLs) such as walking, bathing, or getting dressed, as well as musculoskeletal joint pain, muscle pain, and fever; (2) pulmonary disorders include interstitial lung disease, dyspnea, and reactive airway disease; (3) cardiovascular system problems include chest pain, palpitations (loud or fast heartbeats), chest tightness, myocarditis, heart failure, pericarditis, and orthostatic intolerance, such as postural orthostatic tachycardia syndrome (POTS); (4) neurological impairments include inability to concentrate or focus (commonly referred to as brain fog), memory problems, headaches, olfactory and gustatory dysfunction, sleep difficulties, numbness, tingling sensations, and transient ischemic attack or stroke; (5) gastrointestinal and hepatic disorders include abdominal pain, diarrhea, nausea, and loss of appetite; (6) urology and renal disorders include incontinence, chronic renal disease, and sexual dysfunction; (7) metabolic and endocrine disorders include type 2 diabetes and hypothyroidism; (8) dermatological disorders include skin problems, rashes, and hair loss; (9) mental health conditions include mood swings, anxiety, sadness, post-traumatic stress disorder (PTSD), and psychosis; (10) ear, nose, and throat disorders include olfactory and gustatory loss, earache, and sore throat; and (11) haematology disorders include pulmonary embolism, arterial thrombosis, venous thromboembolism, and other hypercoagulable sequelae and subtypes of PCCs. These symptoms and conditions collectively contribute to the wide-ranging impact of PCCs on individuals' health and quality of life.

Post-COVID-19 Fatigue

Fatigue is a prominent and common symptom in PCCs, irrespective of the severity of the initial acute illness. The prevalence of ongoing fatigue increased from 11.9% at the five-week mark to 93.5% and 92.9% among hospitalized and non-hospitalized patients, respectively, at approximately 79 days (three to four months) after the onset of illness. In comparison, rates of continuing fatigue in ward and ICU patients are 60.3% and 98%, respectively. Even up to seven months after the initial acute illness, around 58% to 60% of cases continue to report persistent fatigue [31].

Post-COVID-19 fatigue refers to COVID-19 patients who have been diagnosed with post-infectious fatigue syndrome (PIFS). Patients were required to experience persistent fatigue for at least six months following recovery to meet the criteria for PIFS, or persistent fatigue symptoms that persisted for at least six weeks after recovery or after an RT-PCR test resulted in a negative result. Prolonged fatigue and constant exhaustion significantly diminish a person's energy, motivation, and ability to concentrate, resulting in a noticeable disability. If fatigue symptoms persist beyond seven months, thorough evaluations and investigations become necessary [32].

The risk factors include older age, female sex, pre-existing autoimmune disease, elevated antinuclear antibodies (ANA), a higher level of vitamin D or ferritin, pre-existing comorbidities such as diabetes mellitus and hypertension, and pre-existing depression or anxiety. Moreover, numerous biological or physical dysfunctions (such as genetic factors) may be the cause. The longer the duration of the acute phase (or of the disease recovery) and the more severe the disease, the higher the risk of suffering from fatigue. Interestingly, in people who have persistent fatigue, there is no association between pro-inflammatory markers and long-term weariness [33].

Various central, peripheral, psychological, and other factors are believed to contribute to the development of fatigue in PCCs. These include direct viral neuro-invasion; miscommunication between cell-mediated immune systems, systemic inflammation, and inflammatory response pathways; adverse effects on psychology and society brought on by the COVID-19 pandemic; immune system malfunction as the likelihood of an autoimmune contribution suggested by the high frequency of pre-existing autoimmune disease and elevated ANA, hormone abnormalities, neurological conditions, and recurrent infection; and peripheral variables that include direct skeletal muscle infection with SARS-CoV-2, which destroys muscle fibers, weakening, and inflammation of neuromuscular connections.

Central factors, such as decreased metabolism in the CNS's frontal lobe and cerebellum, congestion of the lymphatic system, and accumulation of toxic materials as a result of reduced cerebrospinal fluid outflow resistance through the cribriform plate, may be brought on by damage to the olfactory nerve. External factors that cause weakness include musculoskeletal disorders and inflammation of the muscle fibers and neuromuscular connections [34].

Currently, there is no universally accepted method for diagnosing fatigue in PCCs. Diagnosis is primarily based on the exclusion of other diseases, e.g., myalgia encephalomyelitis (ME) and chronic fatigue syndrome (CFS), with similar symptoms, which include fatigue, neurological/pain, cognitive dysfunction (neuro-cognitive), psychiatric, neuroendocrine, autonomic, and immune symptoms, prolonged relapse of exhaustion, long symptom durations, reduced daily activity, depression, and post-exertional malaise. The distinction between ME and CFS remains a mystery. Therefore, research into long-term COVID-19 may assist in developing an understanding of ME/CFS and vice versa.

Using a 5-point Likert scale, where 1 represents never and 5 represents always, the patient-reported outcome measurement information system (PROMIS) is used to diagnose fatigue, as is the fatigue assessment scale. The Chalder Fatigue Scale was utilized to generate a total fatigue score ranging from 0 to 4. A score of two or higher is considered indicative of fatigue. The Borg Scale, which ranges from 0 (nothing at all) to 20 (very, very severe, or maximal), is employed to grade the level of fatigue.

Many suggested effective management tools; e.g., for 14 days, the Chinese integrated medicine rehabilitation guidelines say to take Qingjin Yiqi granules (QJYQ), which are made up of 16 herbs that are extracted with water, then concentrated, sprayed dry to become a powder, and finally excipients are added. The mixture is then pelletized using the dry granulation method, and there is evidence of improvement in fatigue and dyspnea without documented adverse events about QJYQ. For 14 days, molecular inhalation of H2 for two hours each day could potentially improve the physical and respiratory functions of patients recovering from COVID-19. Rehabilitation programs, such as hyperbaric oxygen therapy for pulmonary rehabilitation or physical therapy, include aerobic training, strengthening exercises, diaphragmatic breathing techniques, and mindfulness training. Finally, psychotherapeutic techniques like cognitive-behavioral therapy have been proven to be beneficial for individuals experiencing persistent fatigue [35-36].

Post-COVID-19 Musculoskeletal (Skeletal Muscle, Bone, and Joint) Disorders

About 85.7% of patients have at least one musculoskeletal symptom. Within a month of the infection, reactive arthritis and new-onset inflammatory arthritis usually develop. However, following the development of COVID-19 rheumatoid arthritis, after three to six months, 37.5% had myalgia, 5.7% had arthralgia, 6.8% had new-onset backache, and 50% had generalized body aches. Widespread myalgia in the arm, thigh, lower leg, and shoulder girdle. Localized or radicular pain in the knee, hip, ankle, foot, shoulder, elbow, wrist, hand, chest, myalgia, lower back, neck, and difficulties performing ADLs like walking, dressing, and bathing [36].

Risk factors include a longer hospital stay and having chronic diseases. In addition to the aforementioned disorders, myopathy, neuropathy, cardiorespiratory deficits, and a variety of symptoms affecting various body parts, such as nausea, extreme fatigue, dyspnea (shortness of breath), cough, chest pain, brain fog, short-term memory loss, palpitations, excessive bruising, joint pain, sensitivity to light and sound, coagulation problems, neurological, and gastric, all have an impact on physical weakness.

Some proteins, like the ACE2 receptor and the transmembrane protease serine 2 (TMPRSS2), work together to let spike proteins get started. As soon as the receptor binds, TMPRSS2 breaks down the viral spike protein. This reveals a fusion peptide signal that helps the viral and human membranes join together. It causes viral RNA to be released into the cytoplasm. Homeostatic chondrocytes, endothelial cells, smooth muscle cells, pericytes, muscle stem cells, macrophages, B-cells, T-cells, natural killer cells, and myonuclei all express TMPRSS2. In articular cartilage, trabecular bone, composite unenriched cortical, and osteoblast-enriched tissues, proliferative, hypertrophic, and effector chondrocytes express ACE2. These receptors suggest that skeletal muscle, synovium, and cortical bone could be direct sites of the SARS-CoV-2 infection and its likely long-term effects [37].

The SARS-CoV-2 infection induces the cytokines and signaling molecules (TNF-α, chemokine 10 with the C-X-C motif, interferon-gamma, interleukin (IL)-1β, IL-6, IL-8, and IL-17) that have manifest negative consequences on skeletal muscle, such as decreased protein synthesis and fiber proteolysis. After increasing muscle fibroblast activity, IL-1β and IL-6 may cause fibrosis and platelet hyperreactivity.

Patients with COVID-19 have developed several autoimmune conditions, such as rheumatoid arthritis, psoriatic arthritis, and systemic lupus erythematosus. Corticosteroids are a lifesaving medication in the management of COVID-19, but overuse or long-term use causes many musculoskeletal complications, including osteonecrosis, reduced bone mineral density, avascular necrosis of the hip joint, and osteoporosis with or without fracture [38].

Some of these symptoms can be similar to those in fibromyalgia, post-treatment Lyme disease syndrome, dysautonomia, and MCAS. The post-COVID-19 functional status (PCFS) measure can track functional outcomes over time. A quick and clinically helpful point-of-care technique for identifying PASC early and directing the treatment of orthostatic intolerance is the 10-minute NASA Lean Test. A thorough evaluation of the five COVID-19-related parameters, namely daily living activities, respiratory function, physical function, cognitive function, and quality of life, is crucial. Provocation investigations that include physical, postural, and cognitive challenges are used to find biological variations that are directly related to symptoms that follow COVID-19.

Early mobilization reduces the harmful effects of the infection, especially on muscle mobility. A multidisciplinary rehabilitation approach is crucial to providing ongoing care and support for the recovery process. Virtual rehabilitation programs, including video-linked and online classes, home instruction books, and phone assistance, are likely to become more prevalent shortly [39].

Post-COVID-19 Respiratory Impairments

Regardless of the presence of acute respiratory symptoms or disease severity, dyspnea and breathlessness were reported in approximately 4.6% of patients five weeks after the onset of infection, increasing to 43.4% two months after infection. Approximately 64% of patients three months later still experienced symptoms, and 71% had abnormalities in their lungs. Around one-third of patients exhibit long-term lung damage and functional abnormalities. Elderly individuals, those with acute respiratory distress syndrome (ARDS), prolonged hospital stays, and pre-existing lung abnormalities are more likely to develop fibrotic-like changes in lung tissue.

The virus or its particles remaining in the lungs even after nasopharyngeal swabs yield negative results with COVID-19 recovery: irreversible fibrosis, scarring, and unusual pneumonia. There is a higher chance of pulmonary vascular thromboembolism, with respiratory consequences that last a long time. Even after the infection has cleared, elevated pro-inflammatory cytokine levels (especially IL-6 and TGF-β) lead to lung fibrosis. When SARS-CoV-2 replicates in endothelial cells, the immune system and inflammatory processes inflict serious damage to the lungs and respiratory tract.

The virus gets into the lungs by expressing ACE2 in the upper airway (goblet and ciliated epithelial cells), the lower respiratory tract epithelium (type alveolar), the pulmonary vasculature (arterial smooth muscle), the endothelial cells, and any virus that is still in the lungs after the person has recovered. Cytokine storm activation of the complement system· leads to macrothrombi and microthrombi formation.

Symptoms include chronic cough or shortness of breath (dyspnea), breathlessness, chest pain, reduced exercise capacity, or acute respiratory diseases such as fibrotic lung disease, bronchiectasis, or pulmonary vascular disease. Signs include swollen, congested lungs with alveolitis, round glass opacities, pneumonia, mononuclear inflammatory cells (monocytes and macrophages), fibrinous exudate, and inflammatory edema in the respiratory mucosa and alveolar wall. Platelet-fibrin thrombi, necrotizing bronchiolitis, diffuse alveolar damage, and hyaline membrane formation are also observed [40-44].

Abnormalities in different lung parameters include total lung capacity, forced expiratory volume in the first second, carbon monoxide diffusion capacity, small airway function, and forced vital capacity. In cases of long-term post-COVID-19 breathing difficulties, there may be no signs of irreversible lung damage, while 94% of discharged, asymptomatic cases showed residual lung CT abnormalities. Therefore, it's important to conduct assessments at the time of discharge and one month after symptom onset in hospitalized COVID-19 patients. The long-term clinical significance of imaging and functional abnormalities need further clarification, irrespective of their presence in COVID-19 patients [45-47].

Post-COVID-19 Cardiovascular Impairments 

Approximately 78% of individuals showed abnormal findings in cardiovascular magnetic resonance (CMR) imaging. Among athletes, 60% reported ongoing myocardial inflammation, with myocarditis observed in 15% and past myocardial injuries in 31% of cases.

The prevalence of these abnormalities increased 71 days after the onset of COVID-19 symptoms, irrespective of illness severity, pre-existing conditions, time since diagnosis, or cardiac symptoms. Post-acute COVID-19 infection carries an elevated risk of significant cardiac issues during both the early convalescent phase and the long term.

Direct viral invasion of cardiac tissues via the ACE2 receptor affects various cell types, including cardiac tissue (pericytes, endothelial cells, cardiomyocytes, cardiac fibroblasts, epicardial adipose cells, and vascular cells). Infected cardiomyocytes exhibit sarcomere splitting, enucleation, transcriptional changes, and strong immune reactions. Hyperactive inflammation and coagulation abnormalities contribute to thrombotic consequences. Cytokine storms, hyperinflammation, and endothelial dysfunction lead to leucocyte infiltration/formation of the microvascular thrombosis system. Endothelial dysfunction affects myocardial and pericardial integrity, potentially leading to cardiovascular disease and damage to other organs. Dysregulation of the renin-angiotensin system may contribute to sustained cardio-metabolic demand. Histological abnormalities in the heart reinforce the emergence of cardiac consequences in COVID-19 patients with chronic conditions. A blood clot-prone gene can cause a wide range of thromboembolic events and intravascular coagulation that affect many organs. The rate of venous thromboembolism is thought to be less than 5%.

The main symptoms include chest pain, palpitations, ventricular dysfunction, myocarditis, cardiomyopathy, cardiac arrhythmias, etc. Acute COVID-19-related cardiac problems, such as myocardial injury, myocardial ischemia, infarction, and damage, tachyarrhythmias, heart failure, thrombotic complications, drug-induced cardiotoxicity, stress-related Takotsubo cardiomyopathy, and other complications, even in individuals with moderate symptoms, may persist or progress to post-COVID-19-related cardiac problems such as increased cardiometabolic demands, systolic or diastolic dysfunction, ischemic or non-ischemic cardiomyopathy, myocardial fibrosis or scaring, persistent silent myocarditis, premature ventricular contractions, and ventricular fibrillation. Other symptoms include myocarditis in athletes and autonomic dysfunction associated with cardiac symptoms, such as POTS. The signs include increased troponin levels, low-grade myocardial inflammation/hypertrophied cardiomyocytes with inflammatory infiltrates, focal edema, interstitial hyperplasia/fibrosis/degeneration, necrosis, and signs of lymphocytic myocarditis.

Diagnosis includes a high troponin I level, ST-segment, T-wave abnormalities on an electrocardiogram (ECG), and a decline in left ventricular ejection fraction (LVEF) on cardiac imaging, all of which can indicate myocardial damage. The use of CMR imaging is highly recommended and is growing quickly. This is because it can diagnose both acute and chronic myocardial inflammation sequelae and give a more detailed understanding of the pathophysiological phenomenon, which can be used to classify patients based on risk and improve their care. Moreover, T2 mapping helps rule out active inflammation. With an 89% sensitivity, T1 mapping has a high negative predictive value of 92% for identifying inflammation because T1 relaxation time is sensitive to both acute and chronic inflammation.

There is no single effective treatment or specific pharmacological treatment for post-COVID-19-related cardiac problems. Rehabilitation may not be appropriate for patients who have substantial cardiac impairment. Extra caution should be used when addressing those patients for rehabilitation. Patients whose resting heart rate is greater than 100 beats per minute, whose blood pressure is less than 90/60 or greater than 140/90 mmHg, or whose serum oxygen saturation is less than 95% should be excluded [48-50].

Post-COVID-19 Neurological Impairments 

More than one-third of patients (36.4%) experience persistent cognitive impairment and motor abnormalities after COVID-19, indicating a likelihood of developing long-term neurological effects. The following conditions increase the risk of PCCs: obesity, diabetes, hypertension, cardiovascular disease, chronic lung illness, and age over 65. However, the male gender is associated with a higher chance of PCS neurological sequelae, including Guillain-Barré syndrome. However, there is no gender difference in the classical type [51-52].

The proposed SARS-CoV-2 viral invasion involves reaching the blood-brain barrier or penetrating through olfactory nerves. Direct viral damage to the cortex and nearby subcortical areas can result in viral encephalitis. Hematologic effects include edematous changes in alveolar capillaries, fibrin thrombi, perivascular inflammatory infiltrates, brain lesions, hyperemia, demyelination, acute hypoxic-ischemic injury, systemic inflammation, neurodegenerative diseases, and cerebral vascular alterations. Furthermore, persistent neuropathology and inflammation impact the central and autonomic nervous systems, potentially exacerbating existing neurological conditions or giving rise to new ones [53-54].

Neurological symptoms range from anosmia and ageusia to post-infectious sequelae such as Guillain-Barré syndrome, plexopathies, and cranial neuropathies; transverse myelitis; critical illness neuromyopathy/neuropathy; stroke; Parkinsonism; encephalopathy; status epilepticus; encephalitis; Bell's palsy; vestibulocochlear neuritis; opsoclonus; myoclonus syndrome; myopathy; subclinical cognitive impairment; an increased risk of developing Parkinson's disease and Alzheimer's disease; and neuro-ophthalmic symptoms include optic neuritis, cranial nerve palsies, vision disturbances, and visual field abnormalities. Neuropsychiatric abnormalities, including immunological complications, corticospinal tract manifestations, dysexecutive syndrome, convulsions, sleeplessness, and cerebrovascular incidents, pose a threat to cognitive health, functional status, and overall well-being in COVID-19 survivors [55].

Post-COVID-19 Gastrointestinal and Hepatic Impairment 

It's still unclear how frequently it occurs. At six months, 29% of 1,783 COVID-19 survivors in a prospective cohort experienced these symptoms. These symptoms included abnormal liver function (19%), heartburn (16%), diarrhea (11.5%), constipation (11%), abdominal pain (9%), nausea and vomiting (7%), abdominal discomfort (2.3%), and anorexia. However, based on a review of 43 studies and over 18,000 cases, the prevalence was found to be 15% [56].

It is the SARS-CoV-2 virus that directly infects the ACE2 receptors in cholangiocytes, hepatocytes, and bile duct cells in the hepatobiliary system. It also infects small intestinal enterocytes in the digestive tract. The virus can persist in the gut for a long time, which can lead to active or dormant infections, which can lead to hyperactive inflammation and multisystem inflammatory syndrome in children. This is because zonulin breaks down the gut mucosal barrier function. The high frequency of post-infectious neuro-immune disorders and motility-related disorders, along with alterations in intestinal microbial flora causing changes in the microbiome and microbiota not only within the gut but throughout the body, can lead to a decrease in helpful bacteria and a rise in potentially harmful pathogens. Abnormal coagulation, drug-induced damage, coagulation abnormalities, and cytokine storms are characteristics of such changes [57-58].

The main symptoms include diarrhea, appetite suppression, nausea or vomiting, acute liver injury, cholestasis, abdominal pain, gastrointestinal bleeding, and hepatic dysfunction, which are proportional to the severity of COVID-19. The signs include hepatic cell degeneration, multi-focal necrosis, indicative of cirrhosis, biliary plugs in the small bile duct, atypical lymphocytic infiltration in the portal tract, an increased number of portal veins, actuated Kupffer cells, smooth muscle fragmentation of the portal vein/stenosis of the small intestine, segmental dilatation, degeneration, necrosis, and shedding in the gastrointestinal mucosa, inflammatory infiltrates, and gastrointestinal bleeding. The diagnosis includes elevated serum liver biomarkers (aspartate aminotransferase, alanine aminotransferase, and bilirubin) [59].

Post-COVID-19 Psychological Impairments and PTSD 

Even six months after COVID-19, depression and anxiety are present in 26% of cases, while sleeping disorders occur in 23%. The prevalence of PTSD ranges from 5.8% to 20%. The exact mechanisms are unknown, but factors such as direct viral effects, social stigma, corticosteroid treatment, and the immunological response contribute to psychological symptoms in COVID-19 patients and survivors, including ICU-acquired psychological or neurocognitive disorders [30].

Female gender is associated with a higher risk of persistent psychological symptoms, and there is no evidence linking underlying psychiatric or psychological illnesses to COVID-19-related psychological symptoms. Psychological impairments include anxiety, depression, PTSD symptoms, and cognitive impairment. COVID-19 survivors may experience stress disorders, PTSD, ICU-acquired psychological or neurocognitive disorders, and a range of cognitive and mental health impairments affecting memory, delirium, brain function, and emotional well-being [43,50].

Post-COVID-19 Metabolic and Endocrine Impairments

The incidence of new-onset hyperglycemia among hospitalized COVID-19 patients is estimated to be higher, with approximately 35% of the 551 patients experiencing persistent hyperglycemia for at least six months. The SARS-CoV-2 virus can, directly and indirectly, affect multiple endocrine glands in the body. The pancreas secretes insulin, whereas the ovaries and testes produce sex hormones like progesterone and testosterone. The adrenal gland is responsible for the secretion of cortisol, and the thyroid gland is responsible for the secretion of thyroid hormones. The pancreas and thyroid possess ACE2 receptors, whereas the adrenal gland is susceptible to an exaggerated immune response and cytokine storm. Due to their widespread roles in the body, hormone imbalances can result in a wide range of symptoms. Symptoms include defects in lipid and glucose metabolism, leading to persistent metabolic impairments in PASC. This can result in new-onset diabetes and diabetic ketoacidosis, increasing the risk of adverse clinical outcomes, prolonged hospitalization, and higher clinical scores [60-62].

Post-COVID-19 Olfactory Impairment

After one month, olfactory dysfunction ranges between 41.0% and 61.0%. A viral infection of the olfactory neural system in the nose leads to a sudden loss of olfactory function, and a butanol threshold test is used for diagnosis. Management includes a combination therapy approach, which may involve oral prednisolone, *Ginkgo biloba *supplementation, mometasone furoate nasal spray, and olfactory instruction. After one year of follow-up, more than 80% of patients reported subjective improvement. A better prognosis is associated with a longer duration of follow-up and being female [63-65].

Post-COVID-19 Taste Impairment 

Patients with ARDS often experience cognitive impairment and a diminished quality of life, which ranges from 38.1% to 49.0%. Common presentations include hypogeusia (reduced ability to taste) and dysgeusia (distorted sense of taste). The significant expression of SARS-CoV-2 entry receptors in oral epithelial taste bud cells, along with the involvement of sialic acid and toll-like receptors, contributes to the loss of taste due to viral infection and interference with taste-related transport mechanisms. Interventions, such as oral or topical corticosteroids and phosphodiesterase inhibitors, are still being studied, and recovery tends to plateau after two months, with limited subsequent improvement [66-69].

Post-COVID-19 Menstrual Disturbances 

Around 20% of female athletes reported changes in their menstrual cycles following the COVID-19 infection, and 25% experienced menstrual irregularities one month after the infection. Symptoms include decreased menstrual volume and, less frequently, amenorrhoea (absence of menstruation). Not well known but possibly involving direct viral invasion through ACE2 receptors in the ovarian and endometrial tissues: the immune response and effects on the hypothalamic-pituitary-ovarian axis [70-72].

Hidden Long-Term Post-COVID-19 Cognitive Impairments 

Neurological manifestations and impaired cognition are observed in more than 40% of patients at the outset, with over 30% experiencing long-term cognitive impairment. Post-COVID-19, mild brain damage has been reported in a significant number of survivors, including millions worldwide and millions in the United States. The mechanisms involve brain damage caused by oxygen deprivation, increased risk of strokes, and direct viral effects leading to encephalitis, post-COVID-19 mild brain damage, and systemic inflammation that accelerates cognitive decline. The risk factors are older individuals above 70 years of age and COVID-19-infected individuals who are at a higher risk of COVID-19-related stroke. Megakaryocytes found in the brain capillaries of deceased COVID-19 patients have been associated with strokes. The common symptoms include difficulties in sustained attention, pervasive behavioral problems, cognitive problems, psychological issues, and potentially even death. Diagnosis is made through clinical assessments and performance-based cognitive function tests such as the Montreal Cognitive Assessment (MoCA), Mini-Mental State Examination (MMSE), Compass 31, Telephone Interview for Cognitive Status, and Screen for Cognitive Impairment in Psychiatry [73-75].

Post-COVID-19 Urinary System Impairments 

The estimated prevalence rates of acute kidney injury (AKI) vary between 5% and 43% among infected patients, and the need for kidney replacement therapy was a relatively common characteristic of severe COVID-19, occurring up to 25%, especially in patients admitted to the intensive care unit. The risk factors are low socioeconomic status, cases associated with APOL1 gene polymorphisms, especially in patients of African ancestry, and cases of poor access to healthcare. The presentations include AKI, albuminuria, proteinuria, and hemorrhagia [76].

Several pathophysiological mechanisms have been linked to the changes observed in the kidneys after acute COVID-19. Direct viral invasion through high levels of ACE2 in kidney tissue, including the proximal tubule, epithelial cells, glomerular endothelial cells, monocytes, and the kidney's blood vessels, leads to tubular injury, endothelial damage, inflammatory mediators, complement activation, micro- or macrovascular injury, and podocyte injury. Other factors include cytokine storms, systemic hypoxia-activated complement components (C5b-9), widespread damage to the proximal tube, protein exudate in the balloon cavity and thrombus in the capillaries, and non-specific fibrosis with lymphocytic infiltrates, ultimately resulting in acute tubular necrosis [76].

Post-COVID-19 Dermatological System Impairments

The mechanism includes dermatological lesions, perivascular inflammatory infiltrate, capillary thrombosis with diffuse hemorrhage, pauci-inflammatory thrombotic vasculopathy, thrombotic vasculopathy, clotting, and pressure-induced necrosis. The presentation includes hair loss, erythematous rash, dermatitis urticarial, and multiple unique skin characteristics. While papulosquamous eruptions, especially those of Pernio, lasted longer, urticarial and morbilliform eruptions were longer-lasting and contained chickenpox-like vesicles [77].

The comprehensive post-COVID-19 clinical assessment 

Approaches

Prioritize safety by addressing concerns to prevent post-exertion symptom exacerbation and minimize potential risks associated with excessive diagnostic testing. Conduct evaluations considering physical, psychological, and cognitive aspects, as symptoms can relapse, remit, or new symptoms may emerge over time. Adopt a conservative approach to diagnostics within the first 4-12 weeks following the SARS-CoV-2 infection, as investigations may be inconclusive and some patients' symptoms may improve or resolve. If persistent symptoms persist beyond three months, further evaluation is recommended.

Persistent Post-COVID-19 Symptoms: When to Seek Medical Attention in Specific Cases 

Symptoms persist or develop beyond 12 weeks of recovery; symptoms worsen or change; red flags indicating urgent and potentially life-threatening conditions, such as exercise-induced severe hypoxemia, indicators of serious lung disease, cardiac chest pain, systemic inflammatory disorder in children, pulmonary emphysema, pericarditis with effusion, stroke, or renal failure, are present [78-80].

Detailed Assessment of Acute SARS-CoV-2 Infection and Clinical History

A thorough evaluation of the initial onset of the COVID-19 infection, including symptoms and biometric readings such as oxygen saturation, is crucial. Table 3 shows the classification of COVID-19.

**Table 3:** COVID-19 classification SOB: shortness of breath, ICU: intensive care unit, SpO2: oxygen saturation Image Source: Elseidy et al., 2022 [52]

Mild Illness	Moderate illness	Severe illness	Critical illness
Fever, cough, sore throat, malaise, headache, myalgia, nausea, vomiting, diarrhea, loss of taste, and smell)	Evidence of lower respiratory tract Infection during clinical assessment or imaging	Same as before	Respiratory failure, septic shock, and/or multiple organ dysfunction
No SOB OR imaging findings	SOB or positive imaging findings	Lung infiltrates >50%	–
–	SpO2 ≥94%	SpO2 30	Intubated or ICU admitted

It is important to consider potential serious complications such as pulmonary embolism, heart failure, stroke, myocardial infarction, lung fibrosis, neurologic issues, and severe mental health deterioration. Medications, including prescribed treatments, over-the-counter drugs, and alternative therapies, should be reviewed. The patient's social history may provide insight into relevant factors like isolation, economic hardships, pressure to return to work, grief, or disruption of daily routines, which can impact their well-being. Evaluating the history of oxygen saturation is important. Home self-monitoring using a patient diary can provide reassurance, especially if saturation levels persist below 95%. Exertional desaturation tests, under healthcare supervision, may be informative for patients with a resting oxygen saturation of 96% or higher. A drop of 3% or more during exertion warrants further assessment.

Evaluation of Present Symptoms 

Current symptoms should be assessed, as various underlying pathophysiologic processes may cause them. The presentation may be complicated by different factors and may resemble other post-viral conditions. Red-flag symptoms, such as chest pain, should be thoroughly explored. It is important to consider alternative diagnoses, as not all illnesses in recovering patients are necessarily due to post-acute COVID-19. Comorbidities, other infections, or endocrine disturbances should be evaluated [40-41,80-87].

Clinical Assessment 

Blood pressure measurements, including sitting, standing, and lying positions, should be taken based on the person's signs and symptoms, following relevant guidance. Ambulatory pulse oximetry is recommended for individuals with respiratory symptoms, fatigue, or malaise. Orthostatic vital signs should be assessed for cases reporting postural symptoms, dizziness, fatigue, cognitive impairment, or malaise. A comprehensive and holistic assessment is beneficial. Post-exertion malaise, fatigue, and neurological symptoms should be evaluated. Breathlessness assessment and management should be conducted. Dysfunctional breathing should be assessed and managed. Oxygen requirements should be evaluated and managed. Symptom or palliative care should be provided as needed. Rehabilitation needs should be considered, and appropriate referrals should be made. Cognitive function should be assessed, and venous thromboembolic disease should be considered as a potential new diagnosis (Table 4).

**Table 4:** Summary of various assessment tools for evaluating people with PCCs

COVID-19 conditions	Tools
Functional status and/or quality of life tools	EuroQol-5D (EQ-5D), Patient-Reported Outcomes Measurement Information System (PROMIS) (e.g., cognitive function 4a), Post-COVID-19 Functional Status Scale (PCFS), Barthel Index (BI) of less than 60 points (poor score), Functional Independence Measure (FIM), used to investigate the effects of respiratory rehabilitation on respiratory function, activities of daily living (ADL), quality of life (QoL), and psychological status in elderly patients with COVID-19 who had been discharged from hospital identified and identified as the least less biased assessment with greater reliability and validity compared to other instruments (BI or Katz), and Modified Rankin Scale (mRS), which have been widely used as the primary outcome measure for acute stroke. Most of the scales allow quantification of the magnitude of functional independence based on the assistance requirements across different items related to activities or participation during ADL
Exercise capacity tools: a follow-up appointment is essential to ensure the provision of optimal circumstances for testing that facilitate the achievement of optimal performance standards	10 Meter Walk Test (10MWT): 1-minute sit-to-stand test, 2-minute step test, 6-minute walk used to measure aerobic capacity and endurance in people. Values of 6-minute walk distance (6MWD) <330 m are predictors of poor exercise capacity
Balance and fall risk tools	Tinetti gait: Berg Balance Scale consists of the following 14 items related to balance-specific activities used to assess the clinical evaluation tools for evaluating gait and the dynamic aspect of balance balance capability in older adults. The Biodex Stability System (BSS) is one of the tools presently being used to assess body control both dynamic and limit of stability higher in post-COVID-19 elder cases. Both are used to identify the risk of falling in community-dwelling older persons as dizziness is one of the most frequent balance issues in about one-third of patients
Balance assessment tools	Orthostatic heart rate (HR) assessment: tilt-table testing (e.g., for postural orthostatic tachycardia syndrome (POTS))
Respiratory conditions tools	Modified Medical Research Council Dyspnea Scale (mMRC) of a grade less than 2
Neurologic condition tools	Mini Mental State Examination (MMSE) is a screening test and *not* a diagnostic tool less than 25 is considered normal. Montreal Cognitive Assessment (MoCA), Compass 31 (for dysautonomia), neurobehavioral symptom inventory
Rehabilitation needs assessment (e.g., for sleep, mobility, bowel and bladder function, cognition, pain, and daily living activities)	American Academy of Physical Medicine and Rehabilitation’s functional assessments
Psychiatric and psychosocial condition assessment tools, e.g., traumatic bereavement, the risk to oneself and/or others, COVID-19-related life stresses such as debt, unemployment, and relationship issues) and onward referral where required	Patient Health Questionnaire-9 (PHQ-9) for depression, General Anxiety Disorder-7 (GAD-7) for anxiety disorders, Screen for Post-traumatic Stress Symptoms (SPTSS), PTSD Symptom Scale (PSS), Impact of Event Scale-Revised (IESR), PTSD Checklist for DSM-5 (PCL-5), psychosis screen, Hospital Anxiety and Depression Scale (HADS)
Other conditions and tools	Insomnia Severity Index (ISI) and Wood Mental Fatigue Inventory (WMFI), fatigue severity scale, Connective Tissue Disease Screening Questionnaire

Laboratory Investigation

Testing should be customized based on the patient's symptoms and presentation. The timing criteria for testing range from three weeks to several months following the SARS-CoV-2 infection.

For antigen testing for COVID-19 infection, individuals with asymptomatic or mild disease are generally not infectious beyond 9-10 days after symptom onset. In severe cases (hospitalized patients), viral shedding typically ceases after three weeks. It has been observed that viral shedding is not associated with long-term COVID-19. However, healthcare professionals may recommend testing on a case-by-case basis, despite the general lack of infectiousness. It is important to note that the RT-PCR test used for assessing the current infection is not 100% sensitive. For serologic (antibody) testing, these tests can be used to evaluate previous conditions, but it is worth mentioning that 10-20% of asymptomatic cases may not have detectable antibodies. It is important to note that these laboratory tests are not necessary to establish a diagnosis of PCCs. Basic blood tests should be conducted to assess the affected organs or systems (Table 5).

**Table 5:** Recommended tests based on post-COVID-19 signs and symptoms

Type	Includes
Electrolytes and a complete metabolic panel	Metabolic panel and electrolyte measurements high
Complete blood picture	Blood and RBC count: abnormal WBC, platelets: lymphopenia lymphocytes <1500 per mm3 (reduction of all lymphocyte subsets including CD4^+^ and CD8^+^ T-cells, NK, and B-cells)/lymphopenia linked with radiological lesions
Liver function tests	Liver function tests: elevated serum liver biomarkers (aspartate aminotransferase (AST), alanine aminotransferase (ALT), bilirubin)
Inflammatory markers	Erythrocyte sedimentation rate: C-reactive protein (CRP) >0.5 mg/dL; ferritin to evaluate inflammatory and prothrombotic states
Lipid profile	Total cholesterol, triglyceride, high-density lipoprotein (HDL), low-density lipoprotein (LDL), and very low-density lipoprotein (VLDL)
Renal function and kidney function	Urinalysis, measuring serum creatinine, high blood urea nitrogen, proteinuria, hematuria, cystatin C, loss of electrolytes, measuring glomerular filtration rate (GFR), and checking tubular function by measuring β2-microglobulin in the urine are all things that can be done
Thyroid function	Thyroid-stimulating hormone (TSH) and free T4
Vitamin deficiencies	Vitamin D, vitamin B12
Rheumatologically conditions	Antinuclear antibody, rheumatoid factor, anti-cyclic citrullinated peptide, anti-cardiolipin, and creatine phosphokinase/high levels of CRP/high levels of IL-6
Myocardial injury	Troponin T >14 ng/L
Coagulation disorders and D-dimer	D-dimer >0.5 mg/mL. High D-dimer level. Features of a pro-coagulant state and/or disseminated intravascular coagulation (DIC) include elevated lactate dehydrogenase levels, prolonged prothrombin and partial thromboplastin times, thrombocytopenia, and the occurrence of deep vein thrombosis or pulmonary embolism
Others	Lactate dehydrogenase >250 U/L; CK >170 U/L; interleukin-6 (IL-6); N-terminal (NT); blood sugar
Differentiate symptoms of cardiac versus pulmonary origin	B-type natriuretic peptide: brain natriuretic peptides >150 mg/L: prohormone BNP (NT-proBNP) (50–75 years old, >900 pg/mL; <50 years old, >450 pg/mL)

Imaging

An X-ray is indicated in patients with mild and improving coughs and breathlessness and may not be necessary unless specifically indicated. However, it may be requested at 12 weeks or earlier if there are ongoing symptoms or abnormal findings on previous chest imaging that suggest a significant respiratory illness. Repeat imaging may be considered in cases of acute COVID-19 with persisting abnormalities. A chest CT scan reveals bilateral, multifocal, and peripheral ground glass opacities with or without consolidation. Additionally, fissures near the visceral pleural surfaces may be observed. This imaging modality provides detailed insights into the extent and nature of pulmonary involvement.

The 12-lead ECG is recommended for patients exhibiting cardiac symptoms. ST-segment and T-wave abnormalities on the ECG can indicate myocardial damage. On cardiac imaging, a drop in LVEF may be visible. The use of CMR imaging is highly recommended and is rapidly gaining prominence. While a normal ECG can offer reassurance, if combined with concerning clinical and blood test findings, abnormal results should prompt further cardiac investigation. Regular monitoring of cardiac health is crucial in managing patients with potential cardiac complications.

Reporting and coding

According to the International Classification of Diseases, Tenth Edition Clinical Modification (ICD-10-CM), the World Health Organization (WHO) has coded the PCCs as described in Table 6 [88].

**Table 6:** Morbidity and mortality coding for COVID-19 in ICD-10 and ICD-11 U09.9: Post COVID-19 condition, unspecified – This code is intended for establishing a link with COVID-19 and should not be used in cases that still present with acute COVID-19. Notably, this code has been unavailable in the United States (US) until now. B94.8: Sequelae of other specified infectious and parasitic diseases – In the US, the Centers for Disease Control and Prevention (CDC) recommends using this code for post-COVID conditions until the U09.9 code undergoes review by the US ICD-10 Coordination and Maintenance Committee.

ICD	Code description
10	An emergency ICD-10 code of "U07.1 COVID-19, virus identified" is assigned to a disease diagnosis of COVID-19 confirmed by laboratory testing. An emergency ICD-10 code of "U07.2 COVID-19, virus not identified," is assigned to a clinical or epidemiological diagnosis of COVID-19 where laboratory confirmation is inconclusive or not available. Both U07.1 and U07.2 may be used for mortality coding and tabulation as the cause of death
11	The code for the confirmed diagnosis of COVID-19 is RA01.0. The code for the clinical diagnosis (suspected or probable) of COVID-19 is RA01.1

Management plans for post-COVID-19

Management recommendations for most outpatient patients with PCCs in primary healthcare settings include the following [89-100]:

Patient Education

Healthcare professionals should inform patients that PCCs are not fully understood and assure patients that support will continue to be provided as new information becomes available. Continuously discuss progress and obstacles, and reevaluate objectives as necessary. Acknowledge that post-COVID-19 symptoms may not always have clear explanations or be proportionate to objective findings and should not be disregarded, even if their cause or expected duration is not fully understood.

Encouraging the Use of Diaries and Calendars

Patients should be encouraged to report any new or changing symptoms or health conditions, as well as any changes in activities or routines. This information, especially about trigger events such as menstruation, foods, exertion (physical and cognitive), treatments, or medications, can provide valuable insight into patients' symptoms and lived experiences for healthcare professionals.

Support for Patients With No Serious Symptoms

Patients with no serious symptoms can benefit from support and reassurance during the natural process of recovery. Healthcare professionals involved in various specialties such as pulmonary, cardiac, sport and exercise medicine, psychological, musculoskeletal, neuro-rehabilitation, and general medicine should offer reassurance based on the patient's medical history and reinforce adaptive behaviors.

Holistic Support for Patients With PCCs

This therapy can be incorporated into both short-term and long-term treatments and often works in conjunction with other therapies. The following resources can provide support: healthcare professionals; peer support resources such as patient support groups and online forums; and support groups that connect individuals and provide support and resources for those affected by COVID-19. Healthcare professionals should consider referring patients to social workers, caseworkers, community health workers, or similarly trained professionals to address material, employment, or other social support needs.

Techniques of Supportive Psychotherapy

Supportive psychotherapy techniques include praising, reassuring, normalizing, encouraging, reframing, advice and teaching, language use, suggestion, and counseling. These techniques aim to provide support, normalize experiences, reinforce accomplishments or positive changes, and help clients develop coping skills.

Symptomatic and Rehabilitative Care

Many PCCs can be managed using well-established symptom management techniques. Treatment approaches may include emotional support, ongoing monitoring, symptomatic medication (e.g., acetaminophen for fever), breathing exercises for cough and shortness of breath, physical and occupational therapy, speech and language therapy, vocational therapy, and referrals to appropriate specialists when specific organ systems are involved or additional care is required. Reestablishing social connections, addressing structural determinants, and focusing on mental health and well-being are also important.

Medical Management and Counseling

Medical management aims to maximize the quality of life and function for post-COVID-19 patients. It is based on presenting symptoms, co-morbidities, and treatment objectives. Careful consideration should be given to FDA-approved over-the-counter medications, supplements, and treatments, weighing their benefits and risks. Transparent goal-setting and collaborative decision-making are essential. Counseling on the six lifestyle pillars, including nutrition, sleep, stress reduction, and adherence to the optimal management of underlying medical conditions, should be provided.

Follow-up plans and referrals

Follow-up visits with healthcare professionals can be scheduled every two to three months, with the frequency adjusted based on the patient's condition and illness progression. The clinician should base the timing of assessments on the patient's needs. Referrals may be necessary for specific organ systems or additional care.

## Conclusions

Post-COVID-19 encompasses a diverse array of symptom clusters that can occur in isolation or in combination, often overlapping with each other. These symptoms can be transient, persistent, or exhibit variation over time, affecting various systems of the body. In addition to the wide range of psychological, physical, and social effects, functional difficulties are also associated with post-COVID-19, significantly impacting the health and quality of life of affected individuals. This review demonstrated that, even in cases where acute infection recovery is achieved, the pandemic emphasizes the need for continuous, thorough follow-up of all COVID-19 patients, including those who were initially thought to be asymptomatic, with routine screening for potential long-term persistent infection.

Therefore, we recommended that researchers studying the long-term consequences of COVID-19 should continue to have access to the databases of large integrated healthcare systems and collaborations that facilitate the quick collection and analysis of clinical, laboratory, and diagnostic data to better understand the effects of the virus on the different body organs, especially the kidney and the nervous system. Further studies are necessary to develop a comprehensive understanding of the pathophysiology, prognosis, and effective management strategies. Translational studies with COVID-19 patients collecting data and biospecimens while they are in the hospital and while they are recovering are needed to fully understand the clinical and biological outcomes and to look into how they work. Future prospective, large-scale research with extended follow-up times is required to evaluate the function of different body organs using a variety of parameters and different management techniques.
